# Supplementary material for: Diversity and Experiences of Radiation Oncologists in Canada: A Survey of Gender Identity, Sexual Orientation, Disability, Race, Ethnicity, Religion, and Workplace Discrimination—A National Cross-Sectional Electronic Survey
Source: Curr Oncol. 2025 Nov 17;32(11):643. doi: 10.3390/curroncol32110643 (PMC12651478; doi:10.3390/curroncol32110643)
Supplement: Supplementary file 1 [file curroncol-32-00643-s001.zip › curroncol-3912929-supplementary.pdf]

Default Question Block

Radiation Oncology Workforce Survey: Understanding the Equity and Diversity Climate Facing Practicing Radiation Oncologists in Canada

Researchers:

Medical Resident / Fellow Investigators:

Study Team:

WHY AM I BEING ASKED TO TAKE PART IN THIS RESEARCH STUDY?

You are being invited to participate in an anonymous research study because you are a current radiation oncologist in Canada. The purpose of this study is to help understand the demographics of the Canadian radiation oncology (RO) physician workforce in terms of education, age, race/ethnicity, gender identity, prevalence of disability and accommodation, sexual orientation and socioeconomic background. We are also interested in understanding how satisfied Canadian radiation oncologists are with their job, how they perceive the culture of their working environment, what kind of mentorship opportunities they have, what research radiation oncologists participate in, how ROs split their time between clinical and administrative duties and if ROs have experienced discrimination or harassment at work. Providing your email address at the end is optional, will not be linked to your individual survey responses, and is only needed to provide you with a gift card as a token of appreciation for participating or to consent to be contacted in the future

about a possible interview study.

HOW MANY PEOPLE WILL TAKE PART IN THIS STUDY?
We are inviting all practicing radiation oncologists across Canada to participate.

WHAT WILL HAPPEN DURING THIS STUDY?
You are invited to participate in an anonymous survey that will explore the current diversity of radiation oncologists in Canada. This survey is powered by Qualtrics and will take place online via the survey link provided. We will also have an optional 45 minute interview component to the study at a later point in time, where we will be seeking individuals to interview about topics related to this survey in more depth. This is to better understand and elaborate on the subject matter we are investigating. You may provide your email address in the noted section at the end of this survey if you are interested in taking part in our interview. As a token of appreciation for participating, you have the option of providing your email address (through a separate website that is in no way linked to your survey responses) for the chance to win 1 of 10, $50 Amazon gift cards. We estimate that it will take approximately 15-20 minutes to finish the survey. Your data will be kept on file for 5 years. We aim to disseminate the results of this research 6 months after the survey is initially sent out. This study should take 6 months to complete and the results should be known in about 8 months. All data will be reported in aggregate and no information that could potentially be linked to you will be individually reported. Should there be results that may reveal an individual’s identity (such an sexual orientation at an institution with very few trainees), these results will be grouped

together with other data to ensure that an individual cannot be identified.

WHAT WILL HAPPEN IF I CHOOSE TO WITHDRAW FROM THE STUDY EARLY?

Your participation is completely voluntary and no personally identifiable information such as your name or specific age is collected. All data is stored on encrypted servers at *** and no individual answers will be shared with any institution that may identify you. Because your responses are not linked to your email address, we will be unable to retract your answers should you choose to withdraw at a later time. Therefore, please do not submit this survey should you choose not to participate in this study.

HOW WILL MY PERSONAL INFORMATION BE KEPT PRIVATE?

No individual answers will be shared with any institution and all identifying information will be reported in aggregate. If you decide to participate, the researchers and study staff will do everything that they can to make sure that this data is kept private/confidential. All survey data will be facilitated via Qualtrics. Data collected during your time in this research study will be de-identified and will be held in a database for future use by other researchers. Any future use of this research data is required to undergo review by a Research Ethics Board. No data that could be used to identify you as an individual will be published. All data where there is a small number of respondents that could be identifying of an individual participant will instead be published in aggregate with other measures to ensure confidentiality. **Thorough consultation was undertaken by the designers of this**

survey with equity, diversity and inclusion researchers to ensure that the sensitive nature of the questions asked, and the data recorded will not be linked or identifiable to any one individual or small group to maintain anonymity. We therefore ask you to answer as honestly and openly as you are comfortable so that we can best understand whom comprises our RO population. All data is stored on encrypted servers and no individual answers will be shared with any institution. Even though the likelihood that someone may identify you from the study data is very small, it can never be completely eliminated. Every effort will be made to keep your information be kept confidential, and to follow the ethical and legal rules about collecting, using and disclosing this information. After the study is done, we will still need to securely store your data that was collected as part of the study. We will keep your data and study records stored for 5 years after the end of the study.

WILL I BE COMPENSATED FOR PARTICIPATING IN THIS STUDY?
As a token of appreciation for participating, you have the option of providing your email address (through a separate website that is in no way linked to your survey responses) for the chance to win 1 of 10, $50 Amazon gift cards.

IS THERE ANY CONFLICT OF INTEREST RELATED TO THIS STUDY?
There are no conflicts of interests that the researchers have to report.

WHO DO I CONTACT FOR QUESTIONS RELATED TO THIS STUDY?
If you have questions about taking part in this study you should talk to the

researchers or co-investigators.

If you have questions about your rights as a participant or about ethical issues related to this study and you would like to talk to someone who is not involved in the conduct of the study, please contact ****

DO YOU AGREE TO PARTICIPATE IN THIS STUDY?
BY PRESSING "YES" YOU CONSENT TO PROVIDING YOUR ANSWERS TO THE RESEARCH TEAM.

- ○ No (terminate study)
- ○ Yes (continue on to study questions)

Role and Demographics

Role and Demographics Questions
Why are we asking? These questions allow us to better understand whom comprises the current radiation oncology workforce to provide context for the collected survey data.

What is your current academic rank?

- ◯ Lecturer
- ◯ Assistant professor
- ◯ Associate professor
- ◯ Full professor
- ◯ No academic appointment

What FTE best describes your employment/position? i.e. 0.5, 1.0

Are you a Canadian Medical Graduate (i.e. obtained your MD degree in Canada) or an International Medical Graduate (MD obtained elsewhere, including the US)?

- ◯ Canadian medical graduate
- ◯ International medical graduate

Was your residency training performed at a Canadian institution?

- ◯ Yes
- ◯ No

Where are you currently working as a radiation oncologist (either in an appointed position or in a locum position)?

- Alberta
- British Columbia
- Manitoba
- New Brunswick
- Newfoundland and Labrador
- Northwest Territories
- Nova Scotia
- Nunavut
- Ontario
- Prince Edward Island
- Quebec
- Saskatchewan
- Yukon

How long have you been practicing as a radiation oncologist?

- Less than 5 years
- 6-10 years
- 11-15 years
- 16 - 20 years

- 21 - 25 years
- 26+ years

How large is the group of radiation oncologists you work with at your workplace?

- 1-5
- 6-10
- 11-20
- 21-30
- 30+

What age bracket do you fall within?

- 25-34
- 35-44
- 45-54
- 55-64
- 65-74
- 75+

What is your marital status?

Why are we asking? This question allows us to better understand how marital status affects experiences and opportunities within the field of Radiation Oncology, and how these change over time in response to any future initiatives aimed at mitigating potential biases and inequities against, and/or providing supports for individuals with a given relationship status.

- ○ Single
- ○ Married / in a domestic relationship
- ○ Divorced/separated
- ○ Widowed
- ○ I prefer not to answer this question

Do you have any children (under the age of 18) or dependents you are responsible for or share care for? If so, how many?

Why are we asking? These questions allow us to better understand how providing care/support for family members – including family members defined as having an impairment – may impact experiences and opportunities within the field of Radiation Oncology, and how these change over time in response to any future initiatives aimed to support caregivers.

	0	1	2	3	4	5	5	6	7	8	9	Not Applicable
Children												☐
Adults												☐

Do any of your parents or guardians have a college/university degree?

- One
- Both
- Neither
- I prefer not to disclose this information

Please indicate your approximate household income when you were a teenager

- Less than $25,000
- $25,000 to $50,000
- $50,000 to $75,000
- $75,000 to $100,000
- $100,000 to $125,000
- $125,000 to $150,000
- $150,000 +
- I prefer not to answer

- ○ I don't know

What degrees have you earned? Please select all that apply.

- ☐ MD or equivalent (eg: MBBS)
- ☐ Masters or equivalent
- ☐ PhD or equivalent
- ☐ JD
- ☐ MBA or equivalent

Gender Identity and Sexual Orientation

Gender Identity and Sexual Orientation

Why are we asking? These questions allow us to better understand how gender identity and sexual orientation affect experiences and opportunities within the field of Radiation Oncology, and how these change over time in response to any future initiatives.

Which of the following best describes your current gender identity/identities? (Select all that apply)

[Agender] – Agender is a person who does not identify themselves as having a particular gender.

[Gender-fluid] - Gender-fluid is a nonbinary gender identity that's not fixed and is capable of changing

over time. [Gender identity] – Gender identity means a person's internal sense of whether they are male, female, both or neither. It is a person's internal, deeply held sense of one's gender, and is not visible to others. Individuals may have more than one gender identity, and it can be fluid over time.

[Non-binary] - Nonbinary gender is an umbrella term to describe any gender identity that does not fit into the gender binary of male and female. Nonbinary gender (also sometimes referred to as genderqueer) people may, for example, identify as having no gender, fall on a gender spectrum somewhere between male and female, or identify as totally outside binary gender identities.

[Transgender] - Transgender refers to individuals whose gender identity or expression differs from societal expectations of the sex they were assigned at birth

[Two-spirit] - Two-spirit is an umbrella term used to describe an Indigenous person who does not identify with colonial gender structures. It may be used to describe gender, sexual, and spiritual identity.

- ☐ Agender
- ☐ Gender fluid
- ☐ Man
- ☐ Non-binary
- ☐ Transgender
- ☐ Two-spirit
- ☐ Woman
- ☐ ________ I self-identify as… (please type response)
- ☐ I do not know
- ☐ I prefer not to answer

Which of the following best describes your sexual orientation(s)? (Select all that apply)

[Asexual] - a spectrum that represents individuals who feel little to no sexual attraction

[Bisexual] - an individual attracted to more than one sex, gender, or gender identity

[Gay] - an individual who is emotionally, sexually, and/or romantically attracted to members of the same gender [Lesbian] - a woman who is physically, sexually, and/or emotionally attracted to another woman or female-gendered person

[Queer] – an umbrella term for the whole non-heterosexual community; queerness intentionally has no single definition beyond “not straight”

[Straight] - an individual who is emotionally, sexually, and/or romantically attracted to members of the opposite gender [Two-spirit] – an umbrella term used to describe an Indigenous person who does not identify with colonial gender structures. It may be used to describe gender, sexual, and spiritual identity

[Pan-sexual] – sexual, romantic, or emotional attraction towards people regardless of their sex or gender identity

- ☐ Asexual
- ☐ Bisexual
- ☐ Gay
- ☐ Lesbian
- ☐ Queer
- ☐ Straight / Heterosexual
- ☐ Two-spirit
- ☐ I do not know / Questioning
- ☐ I prefer not to answer

☐ [ ] I self identify as.... (please type response)

☐ Pansexual

Diverse Abilities in the Workplace and Learning Environment

Diverse Abilities in the Workplace and Learning Environment

Do you view yourself as having a disability where the Accessible Canada Act defines disability as:
"any impairment, including a physical, mental, intellectual, cognitive, learning, communication or sensory impairment - or a functional limitation - whether permanent, temporary or episodic in nature, or evident or not, that, in interaction with a barrier, hinders a person's full and equal participation in society."

○ Yes
○ No
○ I prefer not to answer this question

If so, what do you regard as your disability? (Select all that apply)

☐ Blind/visual impairment
☐ Deaf/hearing impairment

- ☐ Speech/communication disability
- ☐ Mental health disability
- ☐ Mobility/physical disability
- ☐ Emotional disability
- ☐ Cognitive disability
- ☐ A health condition that affects your ability to be a physician
- ☐ Not applicable
- ☐ I prefer not to answer this question
- ☐ ________ I prefer to self-describe, please specify:

Race, Ethnic and Cultural Origins and Religious Beliefs

Race, Ethnic and Cultural Origins/Belongings and Religious Beliefs

Why are we asking? These questions allow us to better understand how being racialized in a society, and how ethnicity and cultural origin affects experiences and opportunities within the field of Radiation Oncology, and how these change over time in response to any future initiatives, particularly those around anti-discrimination.

What is your current citizenship status?

- ○ I was born a Canadian citizen

- I am a Canadian citizen through the immigration process
- I am a permanent resident in Canada
- I am on a Work Visa / Study Visa
- I prefer not to answer this question

What is your primary first language?

- English
- French
- An Indigenous / First Nations language or dialect
- Another language
- I prefer not to answer this question

What language besides English do you currently know and would feel comfortable speaking in simple terms to patients in? (Please type "NA" if not applicable)

The 2016 census defined visible minorities as being a member of one of the following groups. Please select as many categories as you identify with.

- South Asian (eg: Indian, Bangladeshi, Sri Lankan)

- ☐ Chinese
- ☐ Black
- ☐ Filipino
- ☐ Latin American
- ☐ Arab
- ☐ Southeast Asian (eg: Cambodian, Indonesian, Thai)
- ☐ West Asian (eg: Iranian)
- ☐ Korean
- ☐ Japanese
- ☐ First Nations
- ☐ Inuk (Inuit)
- ☐ Metis
- ☐ I prefer not to answer this question
- ☐ __________ Other (please specify):
- ☐ Not a visible minority (White/Caucasian)

What would you consider to be your religious or spiritual affiliation that you currently practice or associate with? Please select all that applies.

- ☐ Atheist / Agnostic / No religious or spiritual affiliation currently practiced
- ☐ Bahá'í Faith
- ☐ Buddhism
- ☐ Christianity (any, including Catholic, Protestant, Evangelical, etc.)

- ☐ Confucianism
- ☐ Hinduism
- ☐ Jainism
- ☐ Judaism
- ☐ Islam
- ☐ Native Spirituality
- ☐ Sikhism
- ☐ Spiritual
- ☐ I prefer not to answer this question
- ☐ ______ Other (please specify):

From your name, physical appearance and/or anything you always wear, are you easily identifiable as a member of a specific religion?

- ○ Definitely yes
- ○ Probably yes
- ○ Probably no
- ○ Definitely no
- ○ Not sure
- ○ I prefer not to answer this question

What religion would people assume you belong to? Select all that applies.

- ☐ Buddhism
- ☐ Christianity (Catholic, Protestant, Anglican, Evangelical, etc.)
- ☐ Confucianism
- ☐ Hinduism
- ☐ Judaism
- ☐ Islam
- ☐ Native Spirituality
- ☐ Sikhism
- ☐ ________ Other (please specify):

Job Perceptions

Job Perceptions

"All in all I feel satisfied with my job"

- ☐ Strongly disagree
- ☐ Disagree
- ☐ Neither agree nor disagree
- ☐ Agree
- ☐ Strongly agree
- ☐ ________ If strongly agree/ disagree, please explain:

How often have you thought about moving to a different institution?

- ☐ Never
- ☐ Once or twice
- ☐ Sometimes
- ☐ Often
- ☐ Many times
- ☐ ______ If often/many times, please explain:

How often have you felt regret about deciding to become a physician?

- ☐ Never
- ☐ Once or twice
- ☐ Sometimes
- ☐ Often
- ☐ Many times
- ☐ ______ If often/ many times, please explain:

Thinking about the past year, how would you rate the culture of respect in your department?

"Culture of respect" refers to the attitudes, behaviors, and standards of your colleagues as related to

access to, inclusion of, and level of respect for individual and group needs, abilities, and potential across the spectrum of diverse backgrounds and identities.

- Excellent
- Very good
- Good
- Adequate
- Poor
- Very Poor

Mentorship and Academics

Mentorship and Academics

A formal mentorship program exists within my department:

- Yes
- No

I currently act as a mentor to a trainee(s) and/or colleague(s):

- Yes – to a trainee(s)
-

- Yes – to both, at least one trainee and colleague
- No

I currently have at least one mentor:

- Yes
- No

It is important I have a mentor with similar demographic characteristics to me:

- Strongly disagree
- Disagree
- Neither agree nor disagree
- Agree
- Strongly agree

How many peer-reviewed publications have you been an author on?

- <5
- 5-10
- 10-25
- 25-50

- 50-100
- >100

Approximately how much of your time is spent doing the following? (Sum to 100%)

Patient Care - Seeing patients or doing work directly related to patient care (including time spent rounding with students or residents when a primary purpose is patient care)	0
Research - Conducting research or performing the administrative duties directly related to that research (include time spent obtaining funding, such as writing grants)	0
Teaching - Doing formal didactic teaching unrelated to your patient care or research activities (e.g., giving lectures for medical school students or residents), or preparing for this teaching	0
Administrative Duties - Performing administrative duties not directly related to your research (e.g. committee work, departmental meetings, etc.)	0
Other Work-Related Tasks - Doing any other work-related tasks not included in the categories above	0
Total	0

Personal Experience with Discrimination / Harassment

Personal Experience with Discrimination

During the past five years, have you ever felt discriminated against because of any of the following while working as a radiation oncologist?

Discrimination is defined as unjust or prejudicial treatment based on the grounds of race, age, gender, sex, and/or other traits or characteristics. Discrimination includes but it is not limited to examples such as inequity in work assignments, evaluations/assessments, distribution of resources/support, compensation, and hiring practices. Discrimination can be direct or indirect, subtle or overt. Please check all that apply.

- ☐ Gender
- ☐ Age
- ☐ Race/ethnicity
- ☐ Sexual orientation
- ☐ National origin
- ☐ Disability
- ☐ Religion
- ☐ Marital status
- ☐ Socioeconomic status
- ☐ Pregnancy, childcare responsibilities, other caretaking responsibilities
- ☐ Level of education (Masters vs Doctoral or other advanced degree)
- ☐ Political view
- ☐ ________ Not listed (Please specify):

- ☐ Not applicable
- ☐ I would rather not disclose this information

How often did you feel that you experienced the above discrimination in the past 5 years while working as a radiation oncologist?

- ○ Never
- ○ Once
- ○ 2 – 4 times
- ○ 5 – 10 times
- ○ Regularly / On an ongoing basis

If you personally experienced discrimination, what was the role of the person(s) who harassed / discriminated against you? Check all that apply.

- ☐ Faculty member
- ☐ Nurse
- ☐ Other allied health professional
- ☐ Resident / Clinical Fellow
- ☐ MD student / Other learner
- ☐ Lab worker
- ☐ Staff (administrative, non-faculty)
- ☐ Patient / Patient family

- ☐ Not applicable
- ☐ Not listed. (Please specify the role, but do not provide a name): ________
- ☐ I would rather not disclose this information

Was the person who harassed / discriminated against you someone in a position to directly affect your academic, and/or professional opportunities?

- ○ Yes
- ○ No
- ○ Not sure
- ○ Does not apply
- ○ I would rather not disclose this information

If you experienced harassment/ discrimination perpetrated by a patient/family members, was it based on the following (select all that apply):

- ☐ Gender
- ☐ Age
- ☐ Race/ethnicity
- ☐ Sexual orientation
- ☐ National origin
- ☐ Disability

- ☐ Religion
- ☐ Other
- ☐ Does not apply

Please rate your level of agreement with the following statement:

"I understand how to and feel comfortable reporting harassment incidents at my workplace"

- ◯ Strongly disagree
- ◯ Somewhat agree
- ◯ Strongly agree
- ◯ Does not apply

Within your institution, is there training provided to address any of the following areas or groups of people and if so what format is the training?

	No training provided	Training is in-person	Training is online	Training is via email/policy update
Sexual harassment	☐	☐	☐	☐
Anti-racism	☐	☐	☐	☐
LGBTQ2+	☐	☐	☐	☐
Aboriginal/Indigenous/First Nations health	☐	☐	☐	☐

	No training provided	Training is in-person	Training is online	Training is via email/policy update
Learner mistreatment	☐	☐	☐	☐
Equity, diversity and inclusion	☐	☐	☐	☐
Other forms of discrimination	☐	☐	☐	☐

Please rate the climate of your primary department (or division, if more relevant) on the following continuum by circling a number.
Climate refers to the attitudes, behaviors, and standards of your colleagues as related to access to, inclusion of, and level of respect for individual and group needs, abilities, and potential across the spectrum of diverse backgrounds and identities.

	1	2	3	4	5	
Racist	○	○	○	○	○	Non-Racist
Homogenous	○	○	○	○	○	Diverse
Non-Sexist	○	○	○	○	○	Sexist
Collaborative	○	○	○	○	○	Individualistic
Cooperative	○	○	○	○	○	Competitive
Homophobic	○	○	○	○	○	Non-Homophobic
Not Supportive	○	○	○	○	○	Supportive

How Can We Improve?

How Can We Improve?

What should oncology departments do to address mistreatment or harassment?

What should oncology departments do to advance equity diversity and inclusion in the workplace?

What should oncology departments do to make faculty hiring practices more equitable?

SUBMIT

Please press the FORWARD button below if you are finished the survey and consent to your answers being used in aggregate in this research study. If you do not wish to submit your answers, please close your browser window now.

Powered by Qualtrics
